# Siderophore-harboring gut bacteria and fecal siderophore genes for predicting the responsiveness of fecal microbiota transplantation for active ulcerative colitis

**DOI:** 10.1186/s12967-024-05419-w

**Published:** 2024-06-24

**Authors:** Jingshuang Yan, Guanzhou Zhou, Rongrong Ren, Xiaohan Zhang, Nana Zhang, Zikai Wang, Lihua Peng, Yunsheng Yang

**Affiliations:** 1 School of Medicine, Nankai University, Tianjin, 300071 China; 2 Microbiota Laboratory and Microbiota Division, Department of Gastroenterology and Hepatology, the First Medical Center, Chinese PLA General Hospital, Beijing, 100853 China; 3 National Clinical Research Center for Geriatric Diseases, Chinese PLA General Hospital, Beijing, 100853 China

**Keywords:** Ulcerative colitis, Fecal microbiota transplantation, Clinical response, Siderophore genes

## Abstract

**Background:**

Predictive markers for fecal microbiota transplantation (FMT) outcomes in patients with active ulcerative colitis (UC) are poorly defined. We aimed to investigate changes in gut microbiota pre- and post-FMT and to assess the potential value in determining the total copy number of fecal bacterial siderophore genes in predicting FMT responsiveness.

**Methods:**

Patients with active UC (Mayo score ≥ 3) who had undergone two FMT procedures were enrolled. Fecal samples were collected before and 8 weeks after each FMT session. Patients were classified into clinical response and non-response groups, based on their Mayo scores. The fecal microbiota profile was accessed using metagenomic sequencing, and the total siderophore genes copy number via quantitative real-time polymerase chain reaction. Additionally, we examined the association between the total siderophore genes copy number and FMT efficacy.

**Results:**

Seventy patients with UC had undergone FMT. The clinical response and remission rates were 50% and 10% after the first FMT procedure, increasing to 72.41% and 27.59% after the second FMT. The cumulative clinical response and clinical remission rates were 72.86% and 25.71%. Compared with baseline, the response group showed a significant increase in *Faecalibacterium*, and decrease in *Enterobacteriaceae*, consisted with the changes of the total bacterial siderophore genes copy number after the second FMT (1889.14 vs. 98.73 copies/ng, P < 0.01). Virulence factor analysis showed an enriched iron uptake system, especially bacterial siderophores, in the pre-FMT response group, with a greater contribution from *Escherichia coli*. The total baseline copy number was significantly higher in the response group than non-response group (1889.14 vs. 94.86 copies/ng, P < 0.01). A total baseline copy number cutoff value of 755.88 copies/ng showed 94.7% specificity and 72.5% sensitivity in predicting FMT responsiveness.

**Conclusions:**

A significant increase in *Faecalibacterium*, and decrease in *Enterobacteriaceae* and the total fecal siderophore genes copy number were observed in responders after FMT. The siderophore genes and its encoding bacteria may be of predictive value for the clinical responsiveness of FMT to active ulcerative colitis.

**Supplementary Information:**

The online version contains supplementary material available at 10.1186/s12967-024-05419-w.

## Background

Ulcerative colitis (UC) and Crohn’s disease, two types of inflammatory bowel disease (IBD), are chronic relapsing inflammatory disorders that affect the gastrointestinal tract. The incidence and hospitalization rates in relation to UC are increasing, particularly in Asian regions [1, 2]. The cause of UC remains unknown; however, it is considered to manifest in genetically predisposed individuals following environmental exposure, while its pathogenesis is closely linked to defects in the intestinal epithelial barrier, dysbiosis of the microbiota, and dysregulation of immune responses [3]. Medications for UC include 5-aminosalicylic acid, glucocorticoids, biologics, and immunosuppressants [4, 5]. However, the chronic and recurrent nature of the disease, coupled with the high incidence of adverse effects of various biologics, complicate clinical drug decision-making [6]. This has prompted ongoing research and development for new drugs and therapeutic approaches [7].

Fecal microbiota transplantation (FMT) involves the transfer of fecal microbiota from healthy donor individuals to the gastrointestinal tract of patients to rectify dysbiosis [8]. FMT is an important treatment for recurrent *Clostridioides difficile* (*C. difficile)* infections, with an efficacy rate exceeding 90% [9]. FMT has showed high efficacy and safety in treating active UC, with the clinical remission rates range from 20 to 50%, and clinical response rates within the range of 50%–65% [10–12]. However, partial patients remain unresponsive to FMT. Clinical response rates to FMT in UC vary across studies owing to differences in donor selection, transplantation methods, and other factors [13–17]. Factors such as donor microbiota composition [18, 19], donor–recipient enterotype compatibility [20], post-transplantation recipient microbiota colonization [20], and compliance [21, 22] have been shown associated with to FMT efficacy.

The role of FMT in the treatment of UC is yet to be elucidated. One current challenge is in evaluating and predicting the UC patients most likely to respond to FMT treatment [23]. It is essential to evaluate and identify predictive factors for FMT efficacy, especially some biomarkers before treatment, to provide accurate and effective FMT treatment. In a previous study, we reported that recipient gut microbiota composition was associated with clinical response, remission, and relapse rates [15]. One study reported that in patients achieving clinical remission through oral capsule FMT, the abundance of opportunistic pathogens (*Escherichia*-*Shigella*, *Enterobacter*) significantly decreased after FMT treatment compared with baseline [17]. Paramsothy et al. reported an increased abundance of *Escherichia* species and an enrichment of pathways related to bacterial iron metabolism in patients with UC who did not achieve remission after FMT [19]. Therefore, gut opportunistic pathogens and their virulence factors in predicting FMT efficacy need to be studied.

Siderophore is an important virulence factor (VF) for the survival and proliferation of bacteria, especially pathogenic bacteria. Gram-negative pathogens (*Escherichia coli* [*E. coli*]*, Klebsiella, Shigella, Salmonella*, and *Yersinia*) harbored four common types of siderophores (enterobactin, salmochelin, aerobactin, and yersiniabactin). Enterobactin, encoded by the *entABCDEF-fepABCDG* gene cluster, chelates iron to promote bacterial proliferation, disrupt macrophage iron homeostasis and M1/M2 polarization, and resist host antibacterial effects [24, 25]. Salmochelin, encoded by the *iroBCDEN* gene cluster, is a glycosylated form of enterobactin produced by *Salmonella*, extraintestinal pathogenic *E. coli*, and certain *Klebsiella* species [26]. Aerobactin is a mixed hydroxamate and carboxylate siderophore and a key virulence factor in pathogenic enterobacteria, such as *Klebsiella* and *Yersinia* [27, 28]. Yersiniabactin, present in *Yersinia*, *Klebsiella*, and certain strains of *E. coli*, such as adherent-invasive *E. coli*, utilizes yersiniabactin to grow in competitive environments and may induce inflammation-associated fibrosis [29, 30].

In this study, we investigated the gut microbial variation of patients with active UC during FMT, and further focus on their VFs. Through analyzing the changes of specific taxa and VF-related genes in responders of FMT, we explore the association between siderophore genes and FMT efficacy. And ultimately, we evaluate the potential value of total baseline bacterial siderophore genes copy number as non-invasive biomarkers for predicting FMT responsiveness.

## Methods

### Study design and enrolment

In this single-center, self-controlled cohort study, we investigated patients with active UC who received two FMT procedures at an interval of 8 weeks. Disease assessments were conducted before FMT (F0), 8 weeks after the first FMT (F1), and 8 weeks after the second FMT (F2).

Patients were enrolled on the basis of our previously established inclusion criteria [15, 31] as follows: clinical and colonoscopic confirmation of active UC diagnosis; a Mayo score of ≥ 3; a Mayo endoscopic score of ≥ 2; and either new cases without prior treatment or patients resistant or intolerant to current treatments (including 5-aminosalicylic acid, corticosteroids, immunosuppressants, and biologics). Once exposed to antibiotics, probiotics, or other drugs that affect the gut microbiota, a washout period of at least 1 week was required. All participants signed informed consent. Exclusion criteria comprised patients on long-term prokinetic therapy for diarrhea, those with a history of colectomy or other intestinal surgeries, those with concurrent *C. difficile* or other intestinal pathogen infections, those with severe congenital or acquired immunodeficiency diseases, and those with other severe progressive diseases requiring hospitalization for reasons other than UC. Pregnant or lactating women were also excluded. This study was approved by the Ethics Committee of the PLA General Hospital (S2016-129–01, S2016-130–01), and the Clinical Trial Registry number was ChiCTR-ONH-17012572.

Demographic data concerning the enrolled patients included age, sex, body mass index (BMI), age at disease onset, and disease duration. Disease extent was classified using the Montreal classification [32], and disease activity was assessed on the basis of the Mayo score [33].

### Donor selection and fecal suspension preparation

Based on our previous criteria [15, 31], eligible donors underwent preliminary screening using a questionnaire and laboratory tests. The questionnaire assessed the medical history and lifestyle of the donors, excluding any exposure to infectious pathogens or risky behaviors (such as sexual practices). Donors were serologically screened for the following viruses: HIV; hepatitis A, B, C, and E; syphilis; the Epstein–Barr virus; cytomegalovirus; and rotavirus. Fecal samples were collected to detect intestinal pathogens, including *E. coli* O157, *Salmonella*, *Shigella*, *Staphylococcus aureus*, *Campylobacter*, *Yersinia*, *Vibrio cholerae*, *Vibrio parahaemolyticus*, and *Candida albicans*, toxins A/B of *C. difficile*, and parasites. Additional evaluations included physical examinations, electrocardiograms, chest radiography, urea breath tests, and blood tests to rule out gastrointestinal and non-gastrointestinal diseases. All eligible donors tested negative on the assessments. During the donation period, donors did not use antibiotics, probiotics, or other medications affecting the gut microbiota, had no travel history, and were re-evaluated every three months. Informed consent was obtained from all of the donors.

On the treatment day, donors provided 100–200 g of fresh feces in a sterile container, which was promptly delivered to the laboratory within 1 h. The fecal sample was mixed with 500 mL of sterile saline to produce 300 mL of filtered fecal suspension, which was immediately transferred to the endoscopy center for use. FMT was administered via colonoscopy. The intestines were prepared using 2 L of a bowel-cleansing agent (poly-ethylene glycol electrolyte solution) before treatment. An endoscopic spray tube (AF-2416 PB; Olympus, Japan) was inserted into the ileum through the working channel of the colonoscope, and a total 300 mL of fecal suspension was injected into the spray tube while slowly retracting the scope. After transplantation, patients were advised to rest in bed for at least 45–60 min.

### Measures and outcomes

At three time points (F0, F1, and F2), patients’ clinical symptoms, laboratory parameters, Mayo scores, and endoscopic Mayo scores were recorded. The primary end-points of this study included clinical response, clinical remission, and endoscopic remission.

Clinical response was defined as a decrease in the Mayo score by at least 3 points and at least 30%, along with a decrease in the rectal bleeding sub-score by at least 1 point or a rectal bleeding sub-score of 0 or 1 [34]. Clinical remission was defined as a total Mayo score of ≤ 2 with no individual sub-score > 1. Endoscopic remission was defined as an endoscopic sub-score of 0 or 1 [34]. Consistent with our primary research [15, 31], patients with UC were categorized into clinical response and non-response groups.

### Sample collection and DNA extraction

Fresh fecal samples from patients were collected at three time points (F0, F1, and F2) and stored in 2.0 mL cryovials. We also randomly selected donors’ feces at a certain timepoint. If DNA extraction was not to be immediately performed, all samples were preserved at − 80 °C. DNA was extracted from the fecal samples using the TIANamp Stool DNA Kit (TIANGEN, Beijing, China), following the manufacturer’s instructions. DNA concentration was measured using a NanoDrop One spectrophotometer (Thermo Fisher, Waltham, MA, USA). DNA samples were stored at − 80 °C for subsequent analysis.

### Metagenomic sequencing

DNA extract was fragmented to an average size of about 400 bp using Covaris M220 (Gene Company Limited, China) for paired-end library construction. Paired-end library was constructed using NEXTFLEX Rapid DNA-Seq (Bioo Scientific, Austin, TX, USA). Paired-end sequencing was performed on Illumina NovaSeq ™ X Plus (Illumina Inc., San Diego, CA, USA) at Majorbio Bio-Pharm Technology Co., Ltd. (Shanghai, China) using NovaSeq X Series 25B Reagent Kit according to the manufacturer’ s instructions (www.illumina.com).

The data were analyzed on the free online platform of Majorbio Cloud Platform (www.majorbio.com). Briefly, the raw sequencing reads were trimmed of adapters, and low-quality reads (length < 50 bp or with a quality value < 20 or having N bases) were removed by fastp (https://github.com/OpenGene/fastp, version 0.20.0). Reads were aligned to the human genome by BWA (http://bio-bwa.sourceforge.net. Version 0.7.17) and any hit associated with the reads and their mated reads were removed. The quality-filtered data were assembled using MEGAHIT (https://github.com/voutcn/megahit, version 1.1.2). Contigs with a length ≥ 300 bp were selected as the final assembling result. A non-redundant gene catalog was constructed using CD-HIT (http://weizhongli-lab.org /cd-hit/, version 4.7) with 90% sequence identity and 90% coverage. Gene abundance for a certain sample was eatimated by SOAPaligner (https://github.com/ShujiaHuang/SOAPaligner, version soap2.21release) with 95% identity.

The best-hit taxonomy of non-redundant genes was obtained by aligning them against the NCBI NR database by DIAMOND (http://ab.inf.uni-tuebingen.de/software/diamond/, version 2.0.13), and using VFDB core database to obtain annotation information of VFs.

### Quantitative real-time polymerase chain reaction (qPCR)

The reference plasmid used in this study was a recombinant *E. coli* plasmid containing the *iroB* and *iroN* genes (BGI Tech Solutions, Beijing, China). Plasmid DNA served as a positive control for the salmochelin genes. The standard bacterial strain *Shigella flexneri* (ATCC 12022) was acquired from BIOBW Biotechnology (Beijing, China). Genomic DNA was used as a positive control for the enterobactin and aerobactin genes. The genomic DNA of the *E. coli* LF82 strain was used as a positive control for yersiniabactin genes.

Genomic DNA was extracted from the bacterial strains using the MiniBEST Bacterial Genome DNA Extraction Kit Ver. 3.0 (TaKaRa, Japan). Plasmid DNA extraction was performed using the Fast Plasmid Mini Kit (StarPrep, Beijing, China). Gene sequences for the eight genes were retrieved from the GenBank database, and primers were designed using Primer3Plus software (https://www.primer3plus.com/). Primer specificity was confirmed using the BLAST database (http://www.ncbi.nlm.nih.gov/BLAST). The primers were synthesized using BGI Tech Solutions (Beijing LIUHE) Co., Ltd. Detailed primer information is provided in Additional file 1: Table S1.

All qPCRs were performed using the StepOnePlus system. The 20 µL-qPCR mixture consisted of 10 µL of 2 × TB Green Premix Ex Taq, 0.8 µL of primers, 0.4 µL of ROX, 2 µL of DNA, and 6 µL of ddH_2_O. The thermal cycling conditions were set as follows: (i) 95 °C for 30 s and (ii) 95 °C for 5 s, 60 °C for 30 s, and 72 °C for 1 min, for 40 cycles. Each sample was analyzed in triplicate to ensure quality and reproducibility. Negative controls were reactions without DNA. Standard curves for different qPCR assays were generated using tenfold serial dilutions of genomic DNA from reference strains. All standard curves had an R2 > 0.99, and the amplification efficiencies were between 90 and 110% (Additional file 1: Fig. S1). Positive samples with Ct values < 10 were diluted to achieve Ct values between 10 and 35. The copy number was calculated using the following formula: copies/ng = (ng × 6.023 × 10^−23^)/(template length × 660), assuming a template length equal to the *E. coli* genome base number (4700 kb). qPCR results with Ct values > 35 or those that were Ct negative were considered 0 copies/ng.

### Statistical analysis

Categorical data are presented as numbers and percentages. Continuous data with a non-normal distribution are expressed as medians and interquartile ranges, whereas those with a normal distribution are presented as averages and standard deviations. Statistical analyses were conducted using SPSS v21.0 software (SPSS, Chicago, IL, United States). Categorical data were analyzed using chi-square or Fisher’s exact tests. Continuous data were evaluated using a Mann–Whitney U test and Pearson correlation analysis, whereas paired data were tested using a Wilcoxon rank-sum test. Receiver operating characteristic curves were plotted, and the area under the curve was calculated. Statistical significance was set at P < 0.05.

Alpha diversity was quantified at the operational taxonomic units (OTU) level using the Chao1 index, testing for significant differences with analysis of variance (ANOVA) followed by a Tukey post hoc test. For beta diversity, we performed principal coordinates analysis (PCoA) using Bray–Curtis dissimilarity at the OTU level and determined significant differences among groups using analysis of similarities (ANOSIM). A Wilcoxon rank-sum test was used to determine differences in microbial/functional composition between two groups. Three group-wise comparisons were performed using Kruskal–Wallis testing.

Mappings were created using GraphPad Prism v7.0 (GraphPad Software Inc., CA, United States) and Adobe Illustrator CC 22.0 (Adobe, San Jose, CA, United States) software, and created in the cloud platform of Majorbio Bio-Pharm Technology Co. Ltd.

## Results

### Clinical characteristics of patients with active UC

Seventy patients with confirmed active UC were enrolled between January 2015 and June 2023. All 70 patients underwent two FMT procedures, 58 patients completed the final assessment 8 weeks after the second FMT, and 12 patients only completed the assessment after the first FMT. Of 70 patients, 49 were males and 21 were females. The mean age at onset was 36.20 ± 13.31 years, with an average disease duration of 5.80 ± 6.25 years and a mean BMI of 21.55 ± 3.58. According to the Montreal classification, 5.71% (4/70) of patients had proctitis, 28.57% (20/70) had left-sided colitis, and 65.71% (46/70) had extensive colitis. Thirteen (18.57%), 44 (62.86%), and 13 (18.57%) patients exhibited mild, moderate, and severe activity, respectively. Nine patients did not use any medication during FMT, whereas the remaining patients had a history of medication use, including mesalazine, glucocorticoids, and immunosuppressants (azathioprine) (Table 1).

**Table 1:** Clinical characteristics in the clinical response and non-response FMT groups at baseline

	**Response group**	**Non-response group**	**P-value**
Sex ratio (male/female)	35/16	14/5	0.681
Onset age	36.76 ± 13.560	34.68 ± 12.73	0.672
Disease duration	5.12 ± 5.79	7.63 ± 7.21	0.132
Mayo Score	8.33 ± 2.38	8.00 ± 2.06	0.585
Disease severity			0.16
Mild	10 (19.6%)	3 (15.8%)	
Moderate	29 (56.9%)	15 (78.9%)	
Severe	12 (23.5%)	1 (5.3%)	
Montreal classification			0.307
E1 (proctitis)	3 (5.9%)	1 (5.3%)	
E2 (left-sided colitis)	12 (23.5%)	8 (42.1%)	
E3 (extensive colitis)	36 (70.6%)	10 (52.6%)	
Medication			0.214
None	9 (17.6%)	0	
5-ASA	33 (64.7%)	16 (84.2%)	
Glucocorticoid	8 (15.7%)	3 (15.8%)	
Immunosuppressant	1 (2.0%)	0	

5-ASA, 5 aminosalicylic acid

The donors remained consistent for each patient with UC during the FMT process. In total, 4 donors were included in the study (Additional file 1: Table S2).

### Clinical and endoscopic responses to FMT

After two rounds of FMT, a significant reduction was found in the Mayo scores of patients with UC compared with baseline (8.24 ± 2.29 vs. 4.48 ± 2.66, respectively; P < 0.01). After the first FMT, the clinical response rate was 50% (35/70), with a clinical remission rate of 10% (7/70). After the second FMT, the clinical response rate increased to 72.41% (42/58), and the clinical and endoscopic remission rate increased to 27.59% (16/58). The cumulative clinical response and clinical remission rates were 72.86% (51/70) and 25.71% (18/70).

According to the primary outcome of FMT treatment, the response group comprised 51 patients, and the non-response group comprised 19 patients. No statistically significant differences were observed between the two groups in terms of sex, onset age, disease duration, disease extent and severity, baseline medication use, and donors (Table 1).

### Changes in fecal microbiome with FMT

**Fig. 1:**
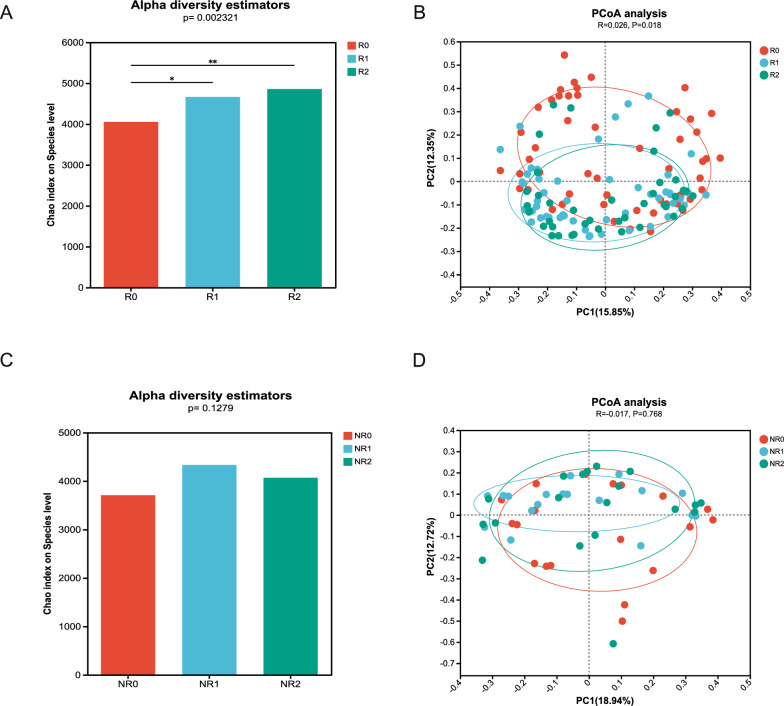
The fecal microbiota diversity changes in the response and non-response groups during FMT. **A** Alpha diversity in the response group, **B** PCoA analysis of the fecal microbiota in the response group, **C** Alpha diversity in the non-response group, **D** PCoA analysis of the fecal microbiota in the non-response group. R0, before FMT in the response group; R1, 8 weeks after the first FMT in the response group; R2, 8 weeks after the second FMT of response group; NR0, before FMT in the non-response group; NR1, 8 weeks after the first FMT in the non-response group; NR2, 8 weeks after the second FMT in the non-response group. FMT, fecal microbiota transplantation; PCoA, principal coordinates analysis

**Fig. 2:**
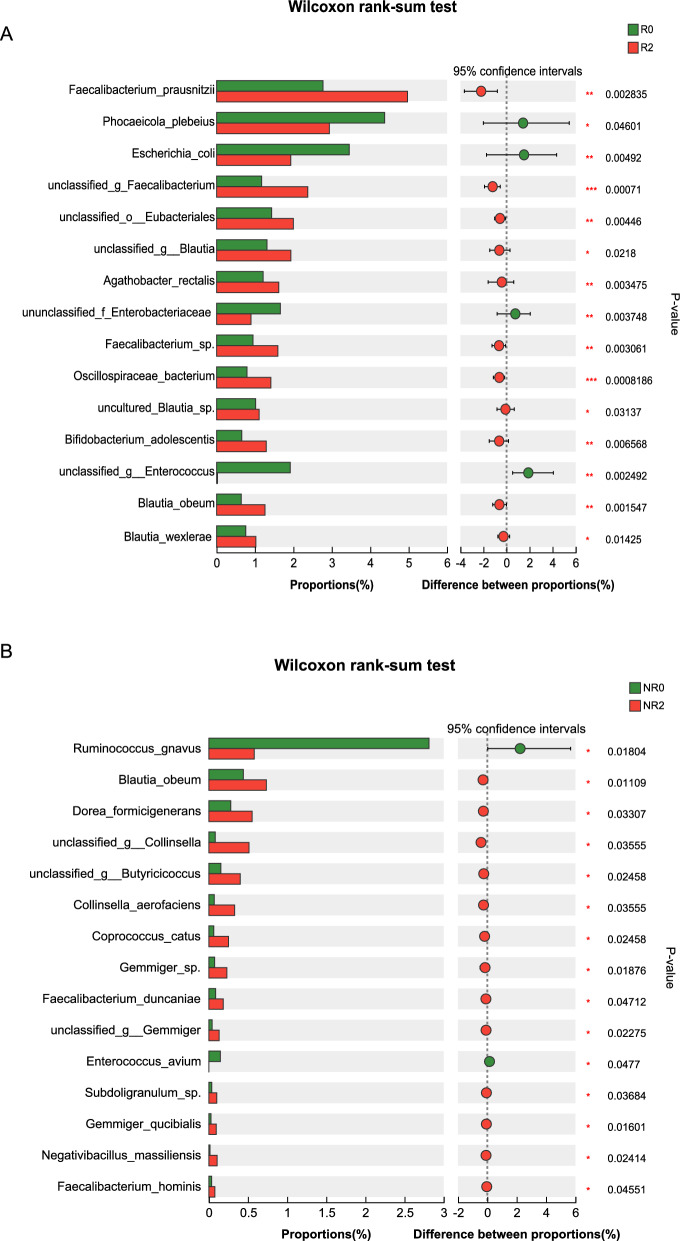
Microbial signatures associated with FMT primary outcomes in species levels. **A** Significant changes in microbial composition in the response group, **B** Significant changes of microbial composition in non-response group. R0, before FMT in the response group; R2, 8-week after the second FMT in the response group; NR0, before FMT in the non-response group; NR2, 8-week after the second FMT in the non-response group. FMT, fecal microbiota transplantation

**Fig. 3:**
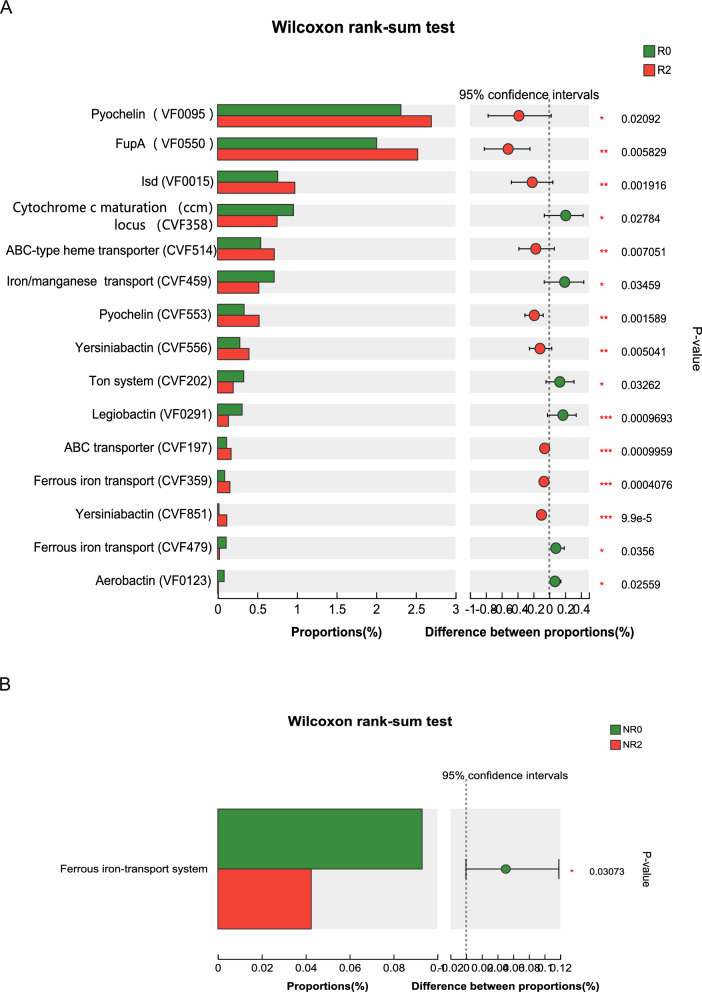
Difference in virulence factors before and after FMT. **A** Specific virulence factors in the response group, **B** Specific virulence factors in the non-response group. R0, before FMT in the response group; R2, 8 weeks after the second FMT in the response group; NR0, before FMT in the non-response group; NR2, 8 weeks after the second FMT in the non-response group. FMT, fecal microbiota transplantation; VFs, virulence factors

In the response group, alpha diversity analysis indicated that microbial richness was lower in patients with UC before FMT, and significantly increased after the first and second FMTs (Fig. 1A). PCoA results suggested that the microbial community composition and structure after FMT differed significantly from that prior to FMT, especially after the second FMT (Fig. 1B). Metagenomic analysis (using a Wilcoxon rank-sum test) indicated a significant increase in the genus abundance of *Faecalibacterium*, *Blautia*, and *Ruminococcus* after the second FMT, whereas *Escherichia* and *Enterococcus* declined significantly (Additional file 1: Fig. S2). Positive changes in the species diversity were shown in the abundance of *Faecalibacterium* (*Faecalibacterium prausnitzii*, *unclassified_g_Faecalibacterium*, *Faecalibacterium sp.*), *Blautia* (*Blautia obeum*, *Blautia wexlerae*, *unclassified_g_Blautia*, *uncultured Blautia sp.*), *unclassified_o_Eubacteriales*, and *Bifidobacterium adolescentis*. In contrast, we observed a decreased richness of *Enterobacteriaceae* (*Escherichia coli*, *unclassified_f_Enterobacteriaceae*), *Phocaeicola plebeius*, *unclassified_g_Enterococcus*, and *Oscillospiraceae bacterium* after the second FMT (Fig. 2A). An analysis of VFs showed that non-specific VFs and regulation of virulence-associated genes in Level 1 were enriched prior to FMT compared with those after the second FMT. Further analysis indicated that iron/manganese transport (CVF459), the Ton system (CVF202), aerobactin (VF0123), and ferrous iron transport (CVF479) were significantly more abundant before FMT (Fig. 3A).

In the non-response group, no obvious changes in microbial diversity and richness were observed in relation to alpha diversity and PCoA analysis results pre- and post-FMT (Fig. 1C, D). The relative abundance of *Ruminococcus gnavus* was lower after the second FMT, whereas *Blautia obeum*, *Dorea formicigenerans*, and *unclassified_g_Collinsella* were increased (Fig. 2B). A VF difference was observed in the ferrous iron-transport system (CVF836), which was enriched prior to FMT (Fig. 3B).

### Baseline microbiome reflecting FMT outcomes

We further analyzed the baseline microbial composition and VFs to determine their association with FMT outcomes. Alpha diversity and PCoA analysis results showed no significant difference in microbial richness and diversity between the clinical response and non-response groups (Additional file 1: Fig. S3, S4). An abundance of bacterial species, namely, *Dorea_sp._CAG:317*, *Amedibacillus dolichus*, *Enterococcus mundtii*, and *Candidatus Egerieimonas faecigallinarum,* were enriched in the response group, whereas *Blautia hansenii*, *Eubacterium sp. CAG:274*, and *Sellimonas intestinalis* were enriched in the non-response group (Additional file 1: Fig. S5). The relative abundance of *E. coli* was slightly higher in the response group, but the difference was not statistically significant.

**Fig. 4:**
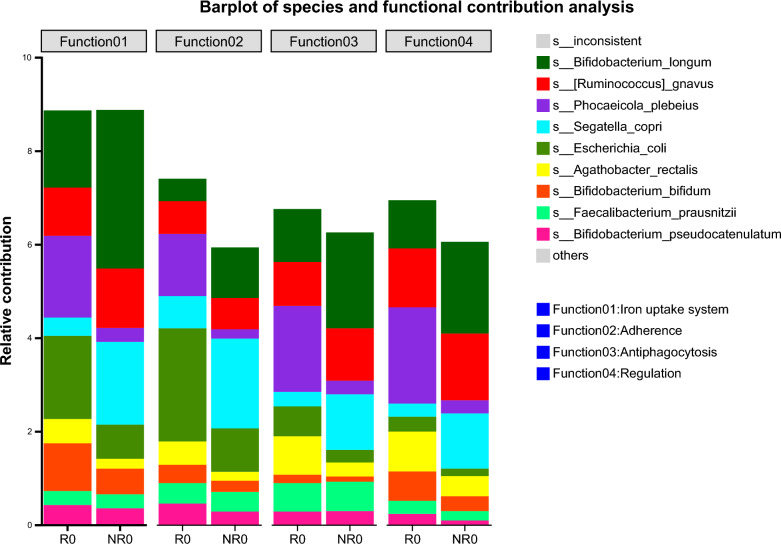
Species and functional contribution analysis between the response and non-response groups. R0, before FMT in the response group; NR0, before FMT in the non-response group

The different VFs between the clinical response and non-response groups were the non-specific VFs (Level 1), especially aerobactin (VF0565, VF0123, VF0229), and iron/manganese transport (CVF459) was enriched in the response group. The functional contribution analysis indicated that *E. coli* and *Phocaeicola plebeius* contributed more to the iron uptake system in the response group than in the non-response group (Fig. 4).

### Variation in the total fecal siderophore genes copy number post-FMT

**Fig. 5:**
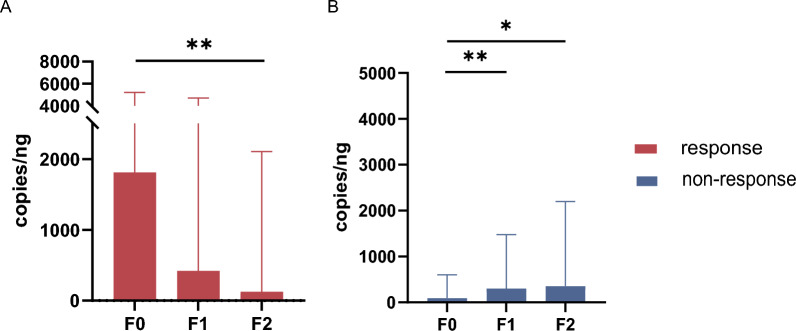
Changes in total fecal siderophore genes copy numbers during FMT. **A** Changes in the total fecal siderophore genes copy number in the response group, **B** Changes in the total fecal siderophore genes copy number in the non-response group. *P < 0.05, **P < 0.01. FMT, fecal microbiota transplantation

Consistent with the variation in the microbiome, the bacterial siderophore genes copy number exhibited a similar tendency. In the response group, the total siderophore genes copy number at baseline was 1889.14 copies/ng (735.68–7469.62), which decreased to 619.86 copies/ng (105.09–7632.16) 8 weeks after the first FMT and further reduced to 98.73 copies/ng (28.21–2001.29) 8 weeks after the second FMT. Compared with baseline, a significant reduction in the total siderophore genes copy number was observed in the response group after the second FMT (P < 0.01) (Fig. 5A). In the non-response group, the baseline total siderophore genes copy number was 94.86 copies/ng (33.28–516.74), which increased to 210.90 copies/ng (58.25–1417.61) 8-week after the first FMT and to 330.78 copies/ng (1.33–1724.43) 8 weeks after the second FMT. Compared with baseline, the total copy number of siderophore genes in the non-response group showed an increasing trend after the second FMT (P < 0.05) (Fig. 5B).

### The association between the baseline total fecal siderophore gene copy number and FMT responsiveness

**Fig. 6:**
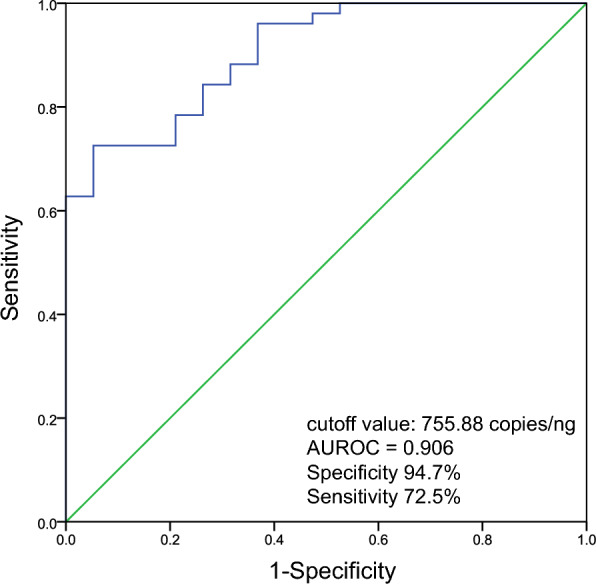
The receiver operating curves for the total copy number of fecal siderophore genes in predicting FMT outcomes. FMT, fecal microbiota transplantation

The baseline total fecal siderophore genes copy number was significantly higher in the response group than in the non-response group (1889.14 vs. 94.86 copies/ng, P < 0.01). Receiver operating characteristic curve (ROC) analysis indicated that the baseline total siderophore genes copy number had an area under the receiver operating characteristic curve of 0.906 (95% confidence interval 0.836–0.976, P < 0.01) to predict the clinical efficacy of FMT (Fig. 6). With a cutoff value of 755.88 copies/ng, specificity, sensitivity, and positive and negative predictive values were 94.7%, 72.5%, 97.4%, and 56.3%, respectively.

## Discussion

Siderophores are crucial for bacterial iron uptake and constitute key VFs for pathogens such as *Klebsiella* and *E. coli*, thereby enhancing their virulence and playing an important role in the infection process [35]. Whether these siderophore genes are related to FMT efficacy has not been studied. In this study, we investigated the changes of gut microbiota and siderophore genes before and after FMT, and explored their association with FMT efficacy.

The results of metagenomics analysis showed that patients with active UC who obtained clinical response of FMT had high abundance of *Enterobacteriaceae,* especially *E. coli*, and siderophore-related VFs at baseline. And FMT shifted the bacterial composition, showing an increase in beneficial bacteria (*Faecalibacterium*, *Blautia*, *Ruminococcus*) and a decrease in pathogenic bacteria (*E. coli*, *unclassified_g_Enterococcus, Phocaeicola_plebeius, Oscillospiraceae_bacterium*). Consistent with our results, high abundance of *Enterobacteriaceae* in active UC had been reported in several studies. He et al.’s study on a Chinese population with UC found a significant increase in the *Proteobacteria* phylum and its *Escherichia*-*Shigella* genus in the feces of patients with active UC, which positively correlated with disease severity [36]. Zhou et al. [37] also reported a marked increase in *Proteobacteria* (*Enterobacteriaceae*) in patients with UC, particularly those with moderate to severe activity. Chen et al. [17] reported that in patients achieving clinical remission through oral capsule FMT, opportunistic pathogens (*Escherichia*-*Shigella*, *Enterobacter*) significantly decreased after treatment, whereas beneficial bacteria (*Faecalibacter* and *Butyricimonas*) increased in abundance. Paramsothy et al. [19] reported that post-FMT responders exhibited an increased abundance of *Eubacterium hallii* and *Roseburia inulinivorans*,. Conversely, non-responders showed an increase in *Fusobacterium gonidiaformans*, *Sutterella wadsworthensis*, and *Escherichia* species. Our results along with previous researches jointly revealed that opportunistic pathogens seemed to had close relationship with FMT outcomes.

Virulence-associated pathway had been reported that they may play an important role in reponse to FMT treatment. Downregulation of the lipopolysaccharide biosynthesis pathway associated with *Enterobacteriaceae* bacteria (*E. coli* and *Klebsiella*) was observed in UC patients achieving clinical remission through oral capsule FMT. In contrast, non-responders showed enhanced lipopolysaccharide biosynthesis pathways [19]. For bacterial iron uptake system, Paramsothy et al. [19] reported non-responders showed enhanced heme biosynthesis pathways. Our results presented that specific VFs associated with a positive therapeutic outcome mainly from iron uptake system, most of them are associated with siderophore systems. Consistently, different VFs between response and non-response group were also derived from siderophore genes, which were enriched in response group. High abundance of the siderophore system associated with *s_Escherichia_coli* and *s_Phocaeicola_plebeius* was observed in response group before FMT. To further prove our analysis, we examined the total fecal siderophore genes copy number of UC patients before and after FMT. Bacterial siderophore genes copy number significantly decreased after FMT in clinical response group, whereas it increased in non-response group. Consisted with the metagenomic results, the baseline total fecal siderophore genes copy number was higher in response group than that of non-response group. And a threshold of 755.88 copies/ng can achieve a specificity of 94.7% and sensitivity of 72.5% for predicting FMT responsiveness. From a dual perspective of bacteria and genes, our results showed that post-FMT clinical remission was associated with the eradication of potential or opportunistic pathogens and the enrichment of beneficial bacteria.

This study had several limitations. The number of patients with FMT included in the study was small and lacked multicenter external validation. Future studies should involve larger sample sizes and multicenter collaborations for validation. Moreover, this clinical study did not investigate the role of siderophores in the pathophysiology of UC. Future research should focus on the relationship between siderophores and UC disease processes and investigate the potential of siderophores as therapeutic targets.

## Conclusions

A significant increase in *Faecalibacterium*, and decrease in *Enterobacteriaceae* and the total fecal siderophore genes copy number were observed in responders post-FMT. Patients with UC and a high abundance of siderophore-harboring bacteria showed better FMT outcomes. The siderophore genes and its encoding bacteria may be of predictive markers for the clinical responsiveness of FMT to active ulcerative colitis.

### Supplementary Information

Additional file 1.

## Data Availability

All data generated or analysed during this study are included in this published article and its supplementary information files. The raw sequence data generated in this study have been deposited to the National Genomics Data Center (China Nation Center for Bioinformation) with accession numbers to be released. The datasets generated and analyzed during this study are available from the corresponding authors on reasonable request.

## Abbreviations

BMI: Body mass index
*C. difficile*: *Clostridium difficile*
*E. coli*: *Escherichia coli*
FMT: Fecal microbiota transplantation
IBD: Inflammatory bowel disease
OTU: Operational taxonomic units
qPCR: Quantitative real-time polymerase chain reaction
ROC: Receiver operator characteristic curve
UC: Ulcerative colitis
VF: Virulence factor

## References

[1] Buie MJ, Quan J, Windsor JW (2023). Global hospitalisation trends for Crohn’s disease and ulcerative colitis in the 21st century: a systematic review with temporal analyses. Clin Gastroenterol Hepatol.

[2] Wei SC, Sollano J, Hui YT (2021). Epidemiology, burden of disease, and unmet needs in the treatment of ulcerative colitis in Asia. Exp Rev Gastroenterol Hepatol.

[3] Le Berre C, Honap S, Peyrin-Biroulet L (2023). Ulcerative colitis. Lancet (London, England).

[4] Ko CW, Singh S, Feuerstein JD (2019). AGA clinical practice guidelines on the management of mild-to-moderate ulcerative colitis. Gastroenterology.

[5] Singh S, Allegretti JR, Siddique SM (2020). AGA technical review on the management of moderate to severe ulcerative colitis. Gastroenterology.

[6] Lasa JS, Olivera PA, Danese S (2022). Efficacy and safety of biologics and small molecule drugs for patients with moderate-to-severe ulcerative colitis: a systematic review and network meta-analysis. Lancet Gastroenterol Hepatol.

[7] Hirten RP, Sands BE (2021). New therapeutics for ulcerative colitis. Ann Rev Med.

[8] Ooijevaar RE, Terveer EM, Verspaget HW (2019). Clinical application and potential of faecal microbiota transplantation. Ann Rev Med.

[9] Quraishi MN, Widlak M, Bhala N (2017). Systematic review with meta-analysis: the efficacy of faecal microbiota transplantation for the treatment of recurrent and refractory *Clostridium difficile* infection. Aliment pharmacol Ther.

[10] Narula N, Kassam Z, Yuan Y (2017). Systematic review and meta-analysis: fecal microbiota transplantation for treatment of active ulcerative colitis. Inflamm Bowel Dis.

[11] Feng J, Chen Y, Liu Y (2023). Efficacy and safety of faecal microbiota transplantation in the treatment of ulcerative colitis: a systematic review and meta-analysis. Sci Rep.

[12] Shi Y, Dong Y, Huang W (2016). Faecal microbiota transplantation for ulcerative colitis: a systematic review and meta-analysis. PLoS ONE.

[13] Fang H, Fu L, Wang J (2018). Protocol for fecal microbiota transplantation in inflammatory bowel disease: a systematic review and meta-analysis. Biomed Res Int.

[14] Paramsothy S, Kamm MA, Kaakoush NO (2017). Multidonor intensive faecal microbiota transplantation for active ulcerative colitis: a randomised placebo-controlled trial. Lancet (London, England).

[15] Ren R, Gao X, Shi Y (2021). Long-term efficacy of low-intensity single donor faecal microbiota transplantation in ulcerative colitis and outcome-specific gut bacteria. Front Microbiol.

[16] Damman CJ, Brittnacher MJ, Westerhoff M (2015). Low level engraftment and improvement following a single colonoscopic administration of faecal microbiota to patients with ulcerative colitis. PLoS ONE.

[17] Chen Q, Fan Y, Zhang B (2023). Capsulized faecal microbiota transplantation induces remission in patients with ulcerative colitis by gut microbial colonization and metabolite regulation. Microbiol Spectrum.

[18] Vermeire S, Joossens M, Verbeke K (2016). Donor species richness determines faecal microbiota transplantation success in inflammatory bowel disease. J Crohns Colitis.

[19] Paramsothy S, Nielsen S, Kamm MA (2019). Specific bacteria and metabolites associated with response to faecal microbiota transplantation in patients with ulcerative colitis. Gastroenterology.

[20] He R, Li P, Wang J (2022). The interplay of gut microbiota between donors and recipients determines the efficacy of faecal microbiota transplantation. Gut Microbes.

[21] Li Q, Zhang T, Ding X (2020). Enhancing patient adherence to faecal microbiota transplantation maintains the long-term clinical effects in ulcerative colitis. Eur J Gastroenterol Hepatol.

[22] Liu Y, Ji X, Huang Y (2023). Older patients benefit more from sequential courses of washed microbiota transplantation than younger population with ulcerative colitis. Scand J Gastroenterol.

[23] Cheng YW, Fischer M (2020). Faecal microbiota transplantation for ulcerative colitis. Are we ready for primetime?. Gastroenterol Clin N Am.

[24] Raymond KN, Dertz EA, Kim SS (2003). Enterobactin: an archetype for microbial iron transport. Proc Natl Acad Sci USA.

[25] Saha P, Xiao X, Yeoh BS (2019). The bacterial siderophore enterobactin confers survival advantage to Salmonella in macrophages. Gut Microbes.

[26] Müller SI, Valdebenito M, Hantke K (2009). Salmochelin, the long-overlooked catecholate siderophore of Salmonella. Biometals.

[27] Russo TA, Olson R, MacDonald U (2015). Aerobactin, but not yersiniabactin, salmochelin, or enterobactin, enables the growth/survival of hypervirulent (hypermucoviscous) *Klebsiella*
*pneumoniae* ex vivo and in vivo. Infect Immun.

[28] Li C, Pan D, Li M (2021). Aerobactin-mediated iron acquisition enhances biofilm formation, oxidative stress resistance, and virulence of *Yersinia*
*pseudotuberculosis*. Front Microbiol.

[29] Dalmasso G, Nguyen HTT, Faïs T (2021). Yersiniabactin siderophore of Crohn's disease-associated adherent-invasive *Escherichia*
*coli* is involved in autophagy activation in host cells. Int J Mol Sci.

[30] Ellermann M, Gharaibeh RZ, Fulbright L (2019). Yersiniabactin-producing adherent/invasive *Escherichia coli* promotes inflammation-associated fibrosis in gnotobiotic Il10(-/-) mice. Infect Immun.

[31] Wang Y, Ren R, Sun G (2020). Pilot study of cytokine changes evaluation after faecal microbiota transplantation in patients with ulcerative colitis. Int Immunopharmacol.

[32] Satsangi J, Silverberg MS, Vermeire S (2006). The Montreal classification of inflammatory bowel disease: controversies, consensus, and implications. Gut.

[33] Lamb CA, Kennedy NA, Raine T (2019). British society of gastroenterology consensus guidelines on the management of inflammatory bowel disease in adults. Gut.

[34] Inflammatory Bowel Disease Group, Chinese Society of Gastroenterology, Chinese Medical Association (2021). Chinese consensus on diagnosis and treatment in inflammatory bowel disease (2018, Beijing). J Dig Dis.

[35] Holden VI, Bachman MA (2015). Diverging roles of bacterial siderophores during infection. Metallomics.

[36] He XX, Li YH, Yan PG (2021). Relationship between clinical features and intestinal microbiota in Chinese patients with ulcerative colitis. World J Gastroenterol.

[37] Zhou Y, Xu ZZ, He Y (2018). Gut microbiota offers universal biomarkers across ethnicity in inflammatory bowel disease diagnosis and infliximab response prediction. mSystems..

