# Supplementary material for: Siderophore-harboring gut bacteria and fecal siderophore genes for predicting the responsiveness of fecal microbiota transplantation for active ulcerative colitis
Source: J Transl Med. 2024 Jun 24;22:589. doi: 10.1186/s12967-024-05419-w (PMC11194913; doi:10.1186/s12967-024-05419-w)
Supplement: Supplementary file 1 — Additional file 1. [file 12967_2024_5419_MOESM1_ESM.pdf]

Additional file 1

Siderophore-harboring gut bacteria and fecal siderophore genes for predicting the responsiveness of fecal microbiota transplantation for active ulcerative colitis

List of Contents

Table S1. Primers and plasmid used in this study.

Table S2. Donor information and fecal siderophore genes copy number.

Figure S1. Standard curves of eight siderophore genes for absolute quantitative real-time PCR. Including *entF* gene (Fig 1A), *fepA* gene (Fig 1B), *iucA* (Fig 1E), and *iutA* (Fig 1F) using genomic DNA from *Shigella flexneri*, *irp1* gene (Fig 1G) and *fyuA* gene (Fig 1H) using genomic DNA from *E. coli* LF82, and *iroB* (Fig 1C) and *iroN* (Fig 1D) from plasmid DNA from recombinant *E. coli.*

Figure S2. Microbial signatures associated with FMT primary outcomes in genus level of response group.

R0, before FMT of response group; R2, 8-week after the second FMT of response group.

Figure S3. The difference of alpha diversity between response and non-response group.

R0, before FMT of response group; NR0, before FMT of non-response group.

Figure S4. The PCoA analysis of response and non-response group.

R0, before FMT of response group; NR0, before FMT of non-response group.

Figure S5. Different species between response and non-response group.

R0, before FMT of response group; NR0, before FMT of non-response group.

Table S1. Primers and plasmid used in this study

Gene	Primer	Product size (bp)
entF	TGTTAATCACCACCGACGAT	293
	CCACACCGAGACATCAAAAC	
fepA	AACACCAACTCTGACGCTTA	214
	AATCCTGTGTCGCTTTTTCG	
iroB	CAACCATCGGTTTGACAGTG	166
	GACGTAACACCGCCGAGTAT	
iroN	AGGTTATCTGCTCTACTCGA	182
	TATTCTGGTAGTCATTGCGG	
iucA	CATCTCAACCTTCAACAGCG	264
	GCTCAGGGAGAACTTGATCA	
iutA	CTCAACTCTTCACGTACCGA	212
	TCGTGATCTTTACTGCTGCT	
irp1	CATGGCAGGAGGTTCTCGTT	155
	GCGCTGCTGTAAATAGTGGC	
fyuA	CGACGGGAAGCGATGACTTA	204
	GCATGTACGGGTCTGGTGAA	

Table S2. Donor information and fecal siderophore genes copy number.

Donor code	Sex	Age	Total fecal siderophore genes copy number (copies/ng)
Donor 1	Male	15	978.58
Donor 2	Female	8	691.91
Donor 3	Male	26	1752.63
Donor 4	Female	28	11.61

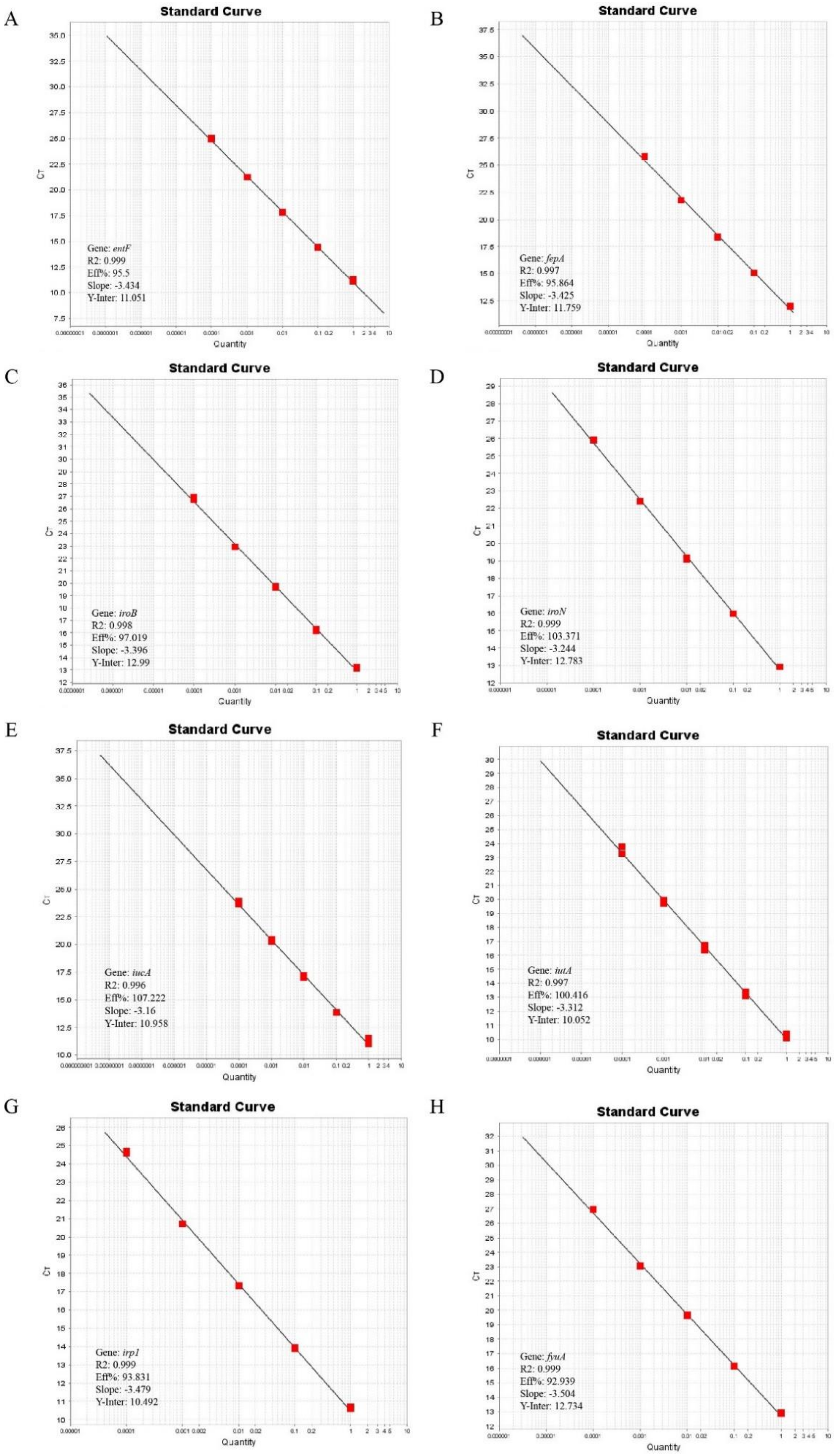

Figure S1. Standard curves of eight siderophore genes for absolute quantitative real-time PCR

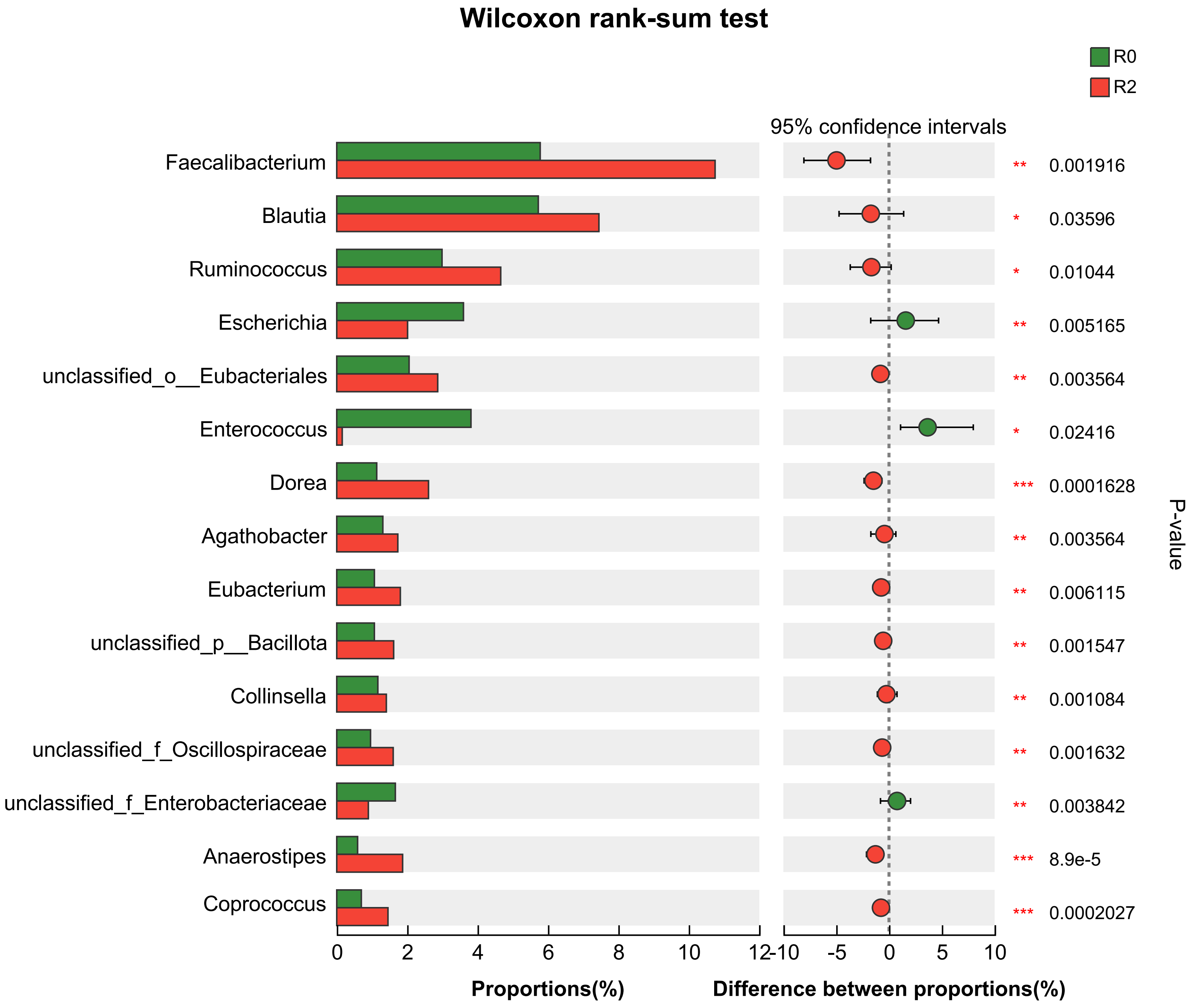

Figure S2. Microbial signatures associated with FMT primary outcomes in genus level of response group

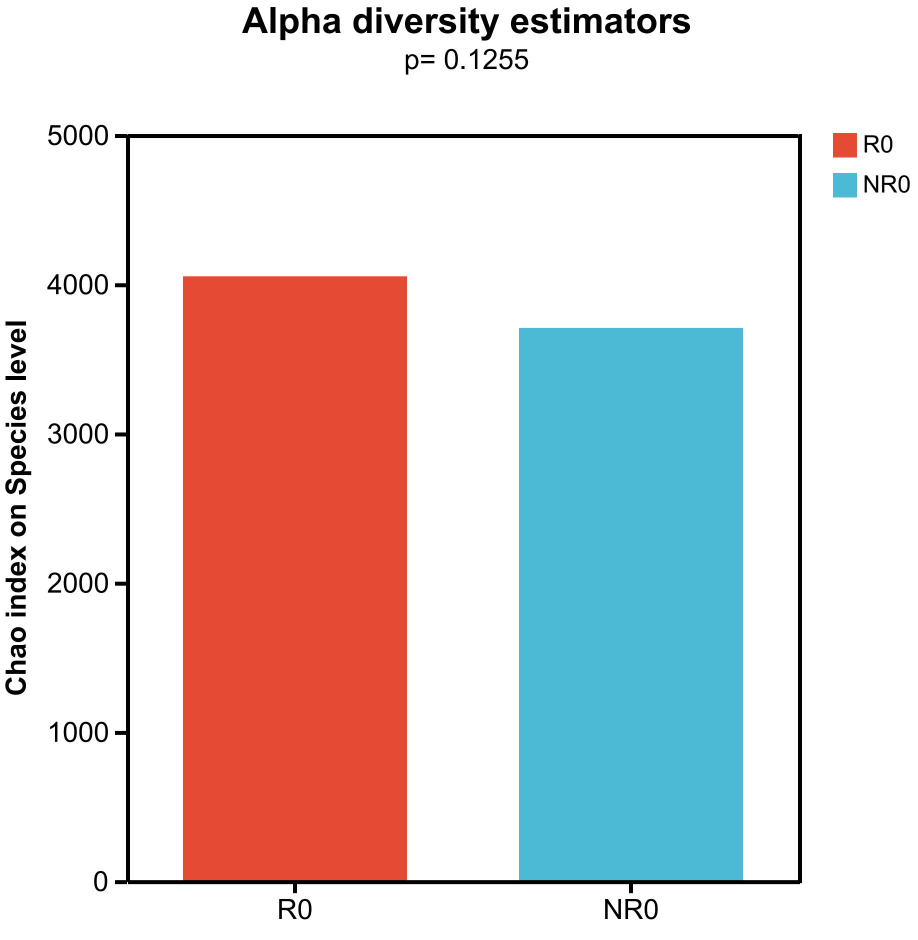

Figure S3. The difference of alpha diversity between response and non-response group

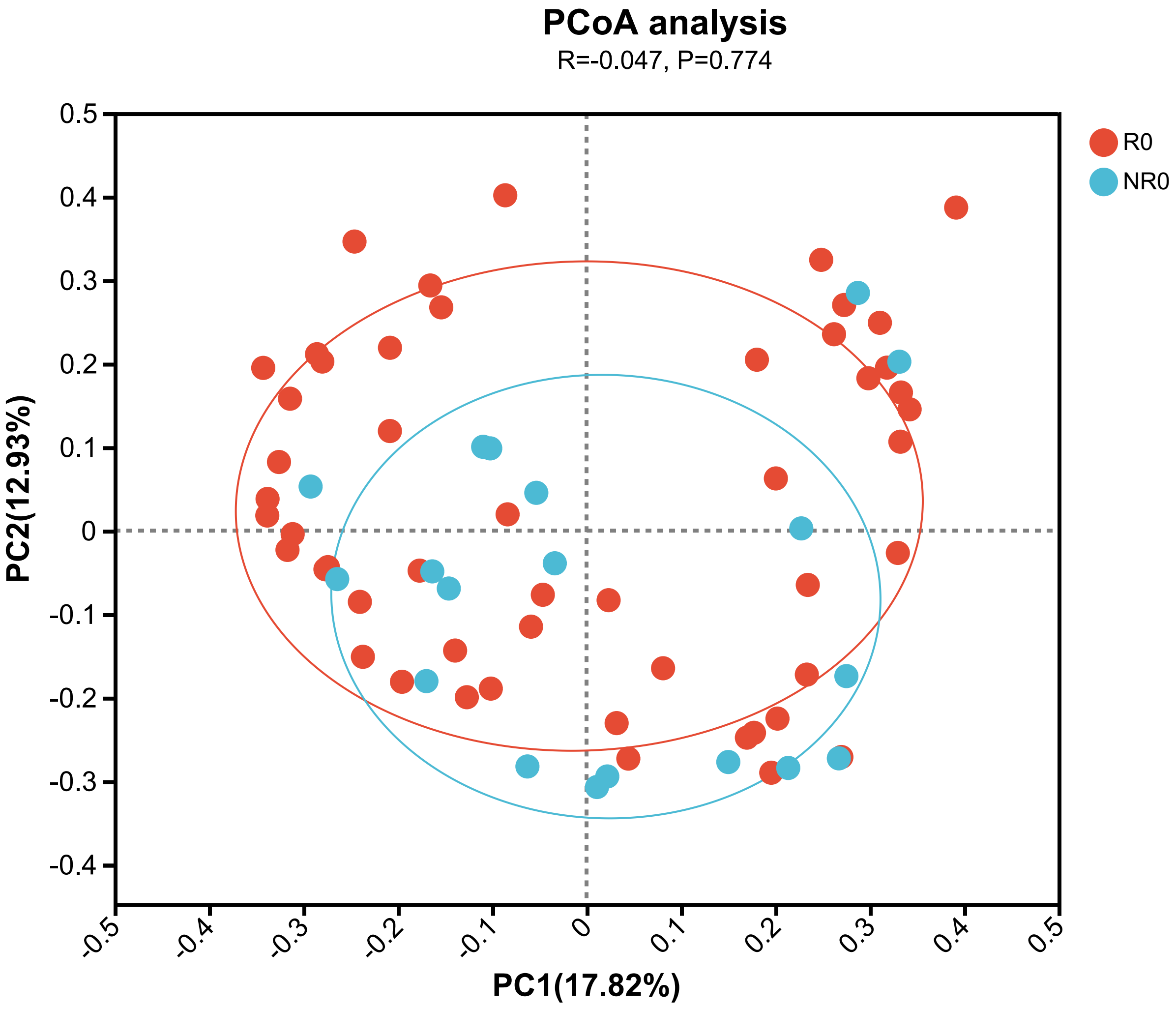

Figure S4. The PCoA analysis of response and non-response group

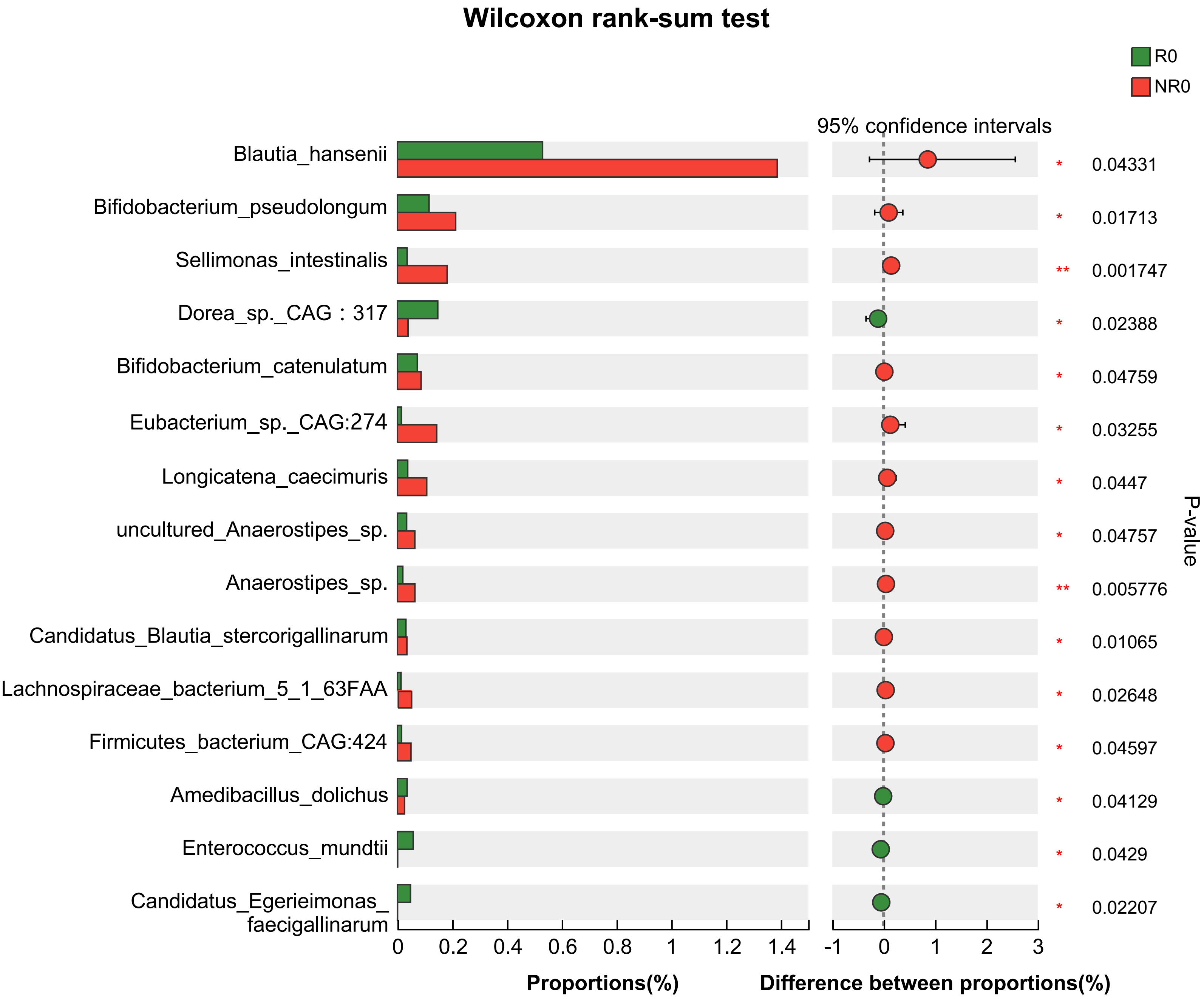

Figure S5. Different species between response and non-response group
